# Microbial load in instruments used in surgeries classified as clean

**DOI:** 10.1186/1753-6561-5-S6-P313

**Published:** 2011-06-29

**Authors:** FMG Pinto, GADA Moriya, KU Graziano

**Affiliations:** 1 Departamento de Enfermagem Médico-Cirúrgica, Nursing School – University of Sao Paulo, Brazil; 2 CSSD, Hospital Alemão Oswaldo Cruz, São Paulo, Brazil

## Introduction / objectives

The number of orthopedic surgery, especially surgery of total hip and knee, have been more frequent due to technological advances. This study aims to determine the microbial load in the instruments used in clean surgeries, quantifying and identifying the genus and species of microbial growth.

## Methods

Orthopedic surgical instruments were immersed, after use, in sterile water, sonicated in ultrasonic washer and consecutively shaken. Then, the lavage was filtered through a 0.45micron membrane, the result was incubated in aerobic medium, anaerobic medium and medium for fungi and yeasts.

## Results

In clean surgeries, results showed that 47% of used instruments had microbiological growth in the range of 1 to 100 CFU/instrument. The most prevalent organism was *Staphylococcus coagulase negative* (28%), followed by *Bacillus subtilis* (11%).This study refuted the hypothesis that clean surgeries happen in micro-organismsfree surgery field.

## Conclusion

The microbiological findings reinforce the importance of antibiotic prophylaxis, practice already well established for this category of surgical procedure.

## Disclosure of interest

None declared.

